# Increased residual cardiovascular risk in U.S. veterans with moderately-elevated baseline triglycerides and well-controlled LDL-C levels on statins

**DOI:** 10.3389/fcvm.2022.982815

**Published:** 2022-11-04

**Authors:** Sarah Leatherman, Ryan Ferguson, Cynthia Hau, Kelly Harrington, Craig Granowitz, Sephy Philip, Peter Paul Toth, Deepak Bhatt, William Boden

**Affiliations:** ^1^VA Boston Healthcare System, Veterans Health Administration, United States Department of Veterans Affairs, Boston, MA, United States; ^2^School of Public Health, Boston University, Boston, MA, United States; ^3^School of Medicine, Boston University, Boston, MA, United States; ^4^Lexicon Pharmaceuticals, The Woodlands, TX, United States; ^5^Amarin Pharma Inc., Bridgewater, MA, United States; ^6^CGH Medical Center, Sterling, IL, United States; ^7^Brigham and Women's Hospital Heart and Vascular Center, Boston, MA, United States

**Keywords:** triglyceride, low-density lipoprotein, residual risk, cardiovascular events, cardiovascular death

## Abstract

**Background:**

Recent studies have demonstrated a causal role for elevated triglycerides (TG) in incident cardiovascular (CV) events in patients with established coronary heart disease (CHD) and those with CV risk factors alone, particularly diabetes.

**Objective:**

Using a large cohort of U.S. veterans with statin-controlled LDL-C levels (40–100 mg/dL), we explored residual CV risk among patients with elevated baseline TG levels (150–499 mg/dL) vs. those with normal TG levels (<150 mg/dL).

**Methods:**

We identified veterans receiving a statin but not a TG-lowering agent from the VA electronic health records database, from 2010 to 2015. We compared composite CV event rates (MI, stroke, unstable angina, coronary revascularization, and CV death) between the elevated TG and normal TG groups. We stratified the study cohort according to 3 CV risk groups: (1) no diabetes and no prior CV event, (2) diabetes and no prior CV event, and (3) prior CV event. We calculated crude event rates, rate ratios, and event rate ratios adjusted for age, sex, systolic blood pressure, estimated glomerular filtration rate, and weight.

**Results:**

The cohort included 396,189 veterans (predominantly male and white) of whom 109,195 (28%) had elevated TG levels. Those with elevated TG were younger (age 73 vs. 77 years) and had a higher body mass index (31.3 vs. 28.3 Kg/M^2^). The overall composite crude and adjusted rate ratios comparing the elevated and normal TG groups were 1.10 (1.09, 1.12) and 1.05 (1.03, 1.06), respectively. For CV risk groups 1, 2 and 3, the adjusted rate ratios comparing the elevated and normal TG groups were 0.99 (0.96, 1.02), 1.05 (1.02, 1.08), and 1.07 (1.04, 1.10), respectively. An association of increased rate ratios did not hold for fatal events.

**Conclusion:**

Those with elevated TG levels and well-controlled LDL-C on statins showed a modest increase in CV events compared to those with normal TG. Elevated TG levels were associated with increased CV events in patients with established CV disease and with diabetes only, suggesting that elevated TG levels are associated with a similar degree of residual risk in high-risk primary prevention and secondary prevention settings.

## Introduction

While coronary heart disease (CHD) survival has increased appreciably in the past three decades, CHD remains the leading cause of death and disability among both men and women in the Western world (1, 2). Elevated low-density lipoprotein cholesterol (LDL-C) is well-recognized as an independent predictor of CHD risk and major adverse cardiac events (MACE) as well as the principal target for dyslipidemic secondary prevention (3–8). Since the advent and widespread use of statins for both primary and secondary prevention, plasma LDL-C levels have been reduced by 25–55% and CHD event rates by 24–45%, as compared with placebo (4–7, 9, 10). However, morbidity and mortality rates among statin-treated patients still remain approximately two-thirds to three-quarters of those found in placebo-treated patients, even among high-risk individuals in whom LDL-C levels ≤70 mg/dL have been achieved on high-potency statin therapy, with or without ezetimibe (5, 6). Thus, despite the important role of statins as a cornerstone of cardiovascular risk reduction and secondary prevention, substantial residual cardiovascular (CV) risk persists, despite potent therapies for lowering LDL-C, including more recently the use of proprotein convertase subtilisin kexin type-9 (PCSK9) inhibitors (4, 5, 8, 11–18).

Clinical and epidemiological studies have likewise demonstrated that elevated baseline triglycerides (TG) levels are an independent risk factor for increased CHD events, and therefore may represent another important component of residual CV risk beyond LDL-C lowering therapies alone (5, 8, 19). Recent Mendelian randomization studies have similarly supported a causal role for TG in the pathogenesis of CHD events, showing that elevated TG levels are not merely a risk marker, but rather a risk factor and thus potentially modifiable with additional dyslipidemic therapy (20, 21). A persistent, and as yet unanswered, question is whether treatment of moderately-elevated TG levels would decrease incident CV events and, in particular, among patients already receiving LDL-C–lowering therapy with statins, including ezetimibe.

Prior randomized trials of TG-lowering agents (e.g., niacin, fibrates, eicosapentaenoic acid (EPA) + docosahexaenoic acid (DHA) omega-3 mixtures), when co-administered with statins, failed to show incremental clinical event reduction in large prospective, placebo-controlled CV outcome studies, despite showing additional favorable effects on reducing elevated TG levels, (22–25) while subgroup analyses suggested possible benefits of TG-lowering in selected patients with dyslipidemia whose TG levels remained elevated, despite statins (26, 27). Similarly, several prospective clinical trials have sought to evaluate the potential cardioprotective role of raising low levels of high-density lipoprotein cholesterol (HDL-C) with various therapeutic interventions, including fibrates, niacin, omega-3 fatty acids (including various generic fish oil preparations), and cholesterol ester transfer protein (CETP) inhibitors, though the results of these trials have been largely negative or inconclusive (22–25, 28–38).

The robust long-term clinical findings of the REDUCE-IT trial showed significant CV event reduction with a high-dose prescription formulation (4 grams daily) of icosapent ethyl, a highly-purified ester of EPA when co-administered with statins in both primary and secondary prevention cohorts, as compared with statins alone (39–41). Subsequently, two observational research databases (from Kaiser-Permanente and Optum Health) likewise demonstrated increased residual risk of major CV events among subjects with a baseline TG 200–499 mg/dL and well-controlled LDL-C (40–100 mg/dL on statins) (42, 43).

Accordingly, we sought to determine if the prevalence and clinical outcomes among U.S. veterans with either established CV disease, or with multiple risk factors for CHD, who had elevated TG levels (150–499 mg/dL) and well-controlled LDL-C (40–100 mg/dL) on statin therapy would likewise be associated with increased residual CV risk who had a lipid profile similar to that of the REDUCE-IT trial (39) and the Kaiser Permanente and Optum observational data sets (42, 43). Identification of a differential signal of increased CV event rates would have important therapeutic implications for pharmacologic strategies to optimize residual dyslipidemic risk reduction beyond lowering LDL-C alone, and to favorably impact incident CV event rates in veterans with (or at risk for) CHD.

## Methods

We explored the incidence between 2010 and 2015 (up to 5 years) of fatal and non-fatal CV events in patients with moderately-elevated TG levels (150–499 mg/dL) at baseline and well-controlled baseline LDL-C (40–100 mg/dL) on statins in a large national cohort of U.S. veterans.

### Study design

This was an observational retrospective cohort study of VA Healthcare System patients. All data were derived from the VA electronic health record (EHR) and the National Death Index (NDI). The VA Boston Healthcare System Institutional Review Board approved the present study with a waiver of informed consent.

### Data sources

Data for this study were obtained from the medical and administrative data collected and maintained by the United States Department of Veterans Affairs (VA) Corporate Data Warehouse (CDW). This nationwide database covers the entire veteran population that utilizes VA health care services and contains individual information on demographic factors, medical history, clinical risk factors and comorbidities, key laboratory values, procedure codes, and diagnoses (inpatient and outpatient) coded with the ICD-9-CM classification system. Date of death was ascertained from the VA Vital Status File and cause of death was obtained from NDI.

### Cohort

Study subjects included U.S. veterans 18 years or older who had at least one TG measurement between 31 and 499 mg/dL in 2010. If multiple TG measurements were available in 2010, the first was used as the baseline value and the corresponding specimen collection date was considered the index date. The index period was defined as the 6 months before and after the index date to accommodate variable laboratory measurement times and prescription refills inherent in the EHR and routine clinical care. During the index period, patients must have had at least one LDL-C measurement between 40 and 100 mg/dL, two encounters in the VA healthcare system, and an active statin medication prescription. Patients who received a prescription TG-lowering agent (fibrates, niacin, or omega-3 fatty acid products) during the 1 year prior to the index date were excluded. Patients without a baseline LDL-C measurement were excluded from the cohort. Similarly, patients receiving a TG-lowering agent during follow-up were censored at the time of the fill. Subjects were categorized as having normal triglycerides if the index TG at baseline was >30 mg/dL and <150 mg/dL and elevated triglycerides if the index TG at baseline was between 150 and 499 mg/dL. If multiple triglyceride measurements were collected in 2010, subjects were categorized based on which TG group occurred most frequently for that subject during the year.

### Baseline characteristics and medical history

Patient demographics including age, sex, race, and ethnicity were determined as the most frequently reported status for individuals. Baseline height, weight, body mass index (BMI), blood pressure, cholesterol, and laboratory measures were recorded as the clinical measure that was closest temporally to the index date. Patient smoking status was predicted using an EHR-based probabilistic algorithm developed based on CDW data (44). Baseline statin prescription was categorized as low- (simvastatin 5 or 10 mg, fluvastatin 20 or 40 mg, lovastatin 10 or 20 mg, pravastatin 10 or 20 mg, and pitavastatin 1 and 2 mg), moderate- (simvastatin 20 or 40 mg, atorvastatin 10 or 20 mg, rosuvastatin 5 mg, fluvastatin 80 mg, lovastatin 40 or 80 mg, pravastatin 40 or 80 mg, and pitavastatin 4 mg), or high-intensity (simvastatin 80 mg, atorvastatin 40 or 80 mg, and rosuvastatin 10, 20 or 40 mg) (42). Comorbid conditions at baseline were identified with pre-defined ICD-9 codes (Table 1) documented in the EHR as having occurred prior to a subject's index date. A history of diabetes mellitus was defined as either two separate outpatient diagnostic encounters of diabetes or one outpatient diagnosis of diabetes and a prescription diabetes medication (including blood glucose regulation agents, hypoglycemic agents, insulin, and oral hypoglycemic agents) in the 2 years prior to the index date.

**Table 1:** ICD-9-CM and ICD-10 codes to define conditions of interest.

**Diagnosis/procedure**	**ICD-9-CM/ICD-10 codes used**
Cardiovascular death	I20.x–I25.x I26.x, I63.3, I63.4, I63.5, I63.8, I63.9
Diabetes	250.x
Hypertension	362.01–362.07, 401, 401.0, 401.1, 401.9, 402, 402.0, 402.00, 402.01, 402.1, 402.10, 402.11, 402.9, 402.90, 402.91, 403.x, 404.x, 405.x, 437.2
Myocardial infarction	410.x, 412.x
Other acute coronary syndrome	414.8x, 997.1x
Peripheral artery disease	440.2x, 440.3x, 443.9x, 250.7x, 443.81, 0.55, 0.60, 39.29, 39.5, 39.9
Retinopathy	250.50, 362.01–362.07
Revascularization	17.55, 36.04, 36.07, 36.09
Stroke	434.x, 436.x, 433.01, 433.11, 433.21, 433.31, 433.81, 433.91, 434.01, 434.11, 434.91
Unstable angina	411.1x, 413.9x

### Cardiovascular risk groups

Subjects were categorized into one of three CV risk groups. The first risk group consisted of subjects without diabetes or prior CVD event. The second CV risk group included patients with diabetes. The third group consisted of patients with a prior CV event identified by a baseline diagnosis of myocardial infarction (MI), ischemic stroke, acute coronary syndrome, or peripheral artery disease (PAD) based on ICD-9 codes, regardless of diabetes diagnosis or other risk factors.

### Outcomes

The primary objective of this study was to assess rates of MACE outcomes, defined as the composite of non-fatal MI, non-fatal stroke, unstable angina, coronary revascularization, or cardiovascular-related death during the follow-up period. Patient follow-up time was defined as the time from the index date to the first CV event, death or study end date defined as September 30, 2015.

Primary outcome events were identified from VA inpatient encounters coded by ICD-9-CM and ICD-9-PCS and NDI coded by ICD-10 (Table 1). Non-fatal MI was identified by codes 410.x and 412.x. Stroke was defined as any event coded by 434.x or 436.x. Unstable angina was identified by codes 411.1 and 413.9. Coronary revascularization was identified by codes 17.55, 36.04, 36.07 and 36.09. Cardiovascular death was identified by cause of death ICD codes I20-I25, I63.3, I63.4, I63.5, I63.8, and I63.9.

### Statistical methods

Baseline characteristics, vital signs and medical history were analyzed descriptively (mean and standard deviation (SD) for continuous variables and frequency and percent for categorical variables), both for the 3 CV risk groups and by TG cohort. Additionally, patient utilization of hospital services, all-cause hospitalizations, and polypharmacy were summarized by TG cohort.

MACE outcome rates were compared between the elevated TG (150–499 mg/dL) and normal TG (<150 mg/dL) groups. Unadjusted event rates (per 1,000 person years) and corresponding 95% confidence intervals (CIs) were calculated for both groups. Poisson regression was used to calculate rate ratios and 95% CIs adjusted for age, sex, baseline systolic blood pressure, eGFR, and weight. Additionally, adjusted analyses were stratified by CV risk groups, race, and statin intensity. Exploratory analysis to assess TG level as a time-varying exposure over the study period was conducted using Cox regression. All analyses were carried out with SAS version 9.2 (SAS Institute, Cary, NC).

## Results

**Figure 1:**
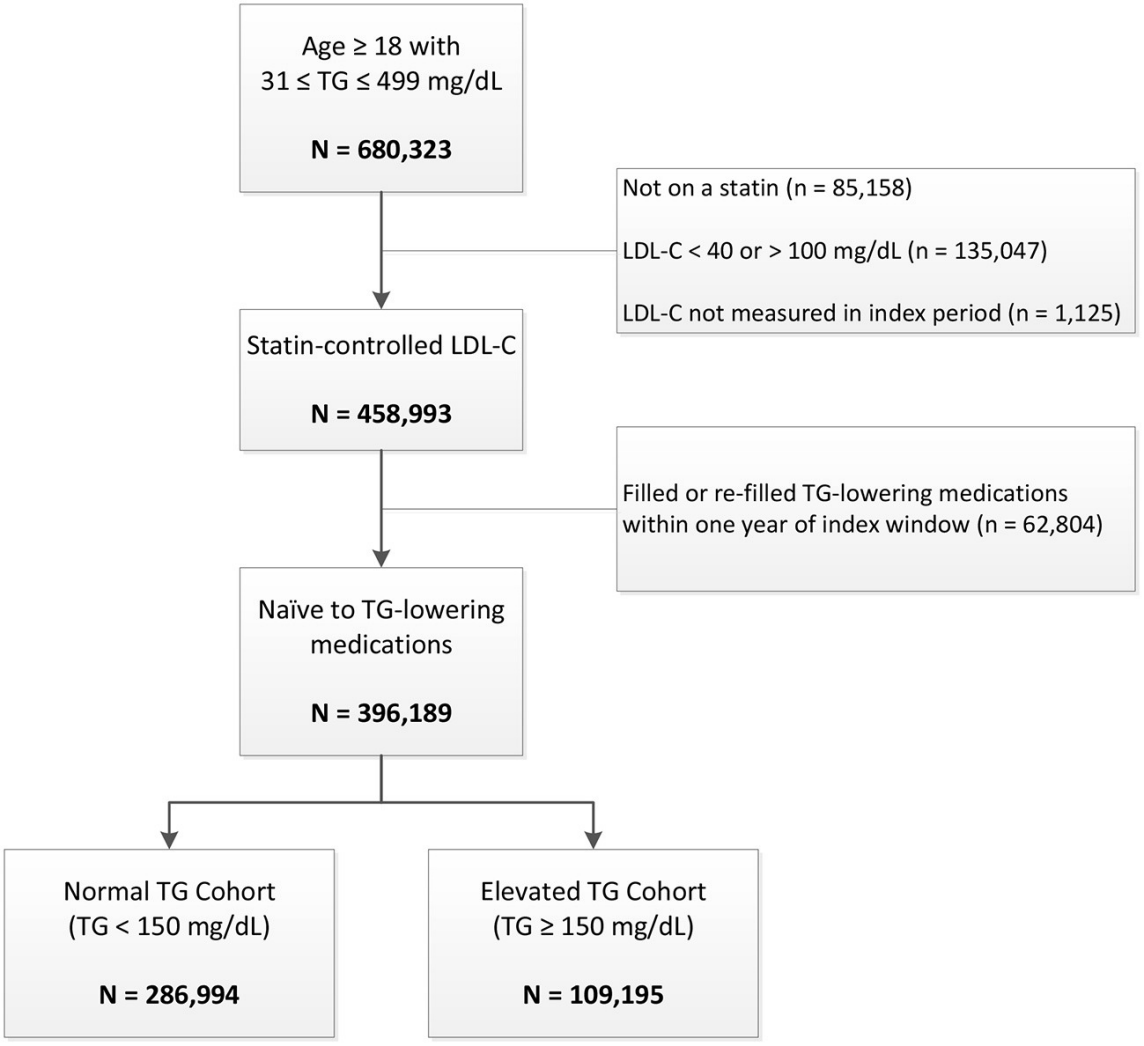
Patient disposition. LDL-C indicates low-density lipoprotein cholesterol; TG, triglycerides.

There were 680,527 veteran patients ≥ age 18 years who had a baseline TG measurement between 31 and 499 mg/dL in 2010 (Figure 1). Of those individuals, 447,580 (66%) had statin-controlled LDL-C levels between 40 and 100 mg/dL. The final analytic cohort included 396,189 veterans naïve to TG-lowering medications, of whom 109,195 (28%) had elevated TG levels at baseline.

### Demographic and baseline clinical characteristics

Subjects with elevated TG levels were younger, more likely to be white, and were more likely to have ever smoked (Table 2). These patients also had higher baseline BMI, weight, blood pressure, total cholesterol, LDL-C, hemoglobin A1c (HbA1c), and more prior CV events than subjects with normal TG at baseline. Subjects with normal TG levels had higher baseline HDL-C and fewer risk factors for future CV events than individuals with elevated TG at baseline. There was no difference between TG groups in patient utilization of hospital services, all-cause hospitalizations, or medication use.

**Table 2:** Demographic and baseline characteristics.

	**Elevated TG**	**Normal TG**	***P*-value**
	**(*n* = 109,195)**	**(*n* = 286,994)**	
**Baseline characteristics** ^0^			
Male (%)	98.2	98.6	<0.0001
Race (%)			<0.0001
White	80.3	74.5	
Black or African American	5.9	10.0	
Asian, Pacific Islander, or other	1.4	1.3	
Multiple race	0.6	0.6	
Unknown	11.8	13.6	
Ethnicity (%)			<0.0001
Hispanic or Latino	3.8	3.5	
Not Hispanic or Latino	88.3	87.2	
Unknown	7.9	9.3	
Age, yrs	73.0 ± 10.3	76.8 ± 9.7	<0.0001
Height, cm	175.4 ± 7.6	175 ± 7.7	0.0001
Weight, kg	95.8 ± 21.1	86.7 ± 19.8	<0.0001
BMI, kg/m^2^	31.2 ± 6.3	28.3 ± 6.0	<0.0001
Smoking status (%)			<0.0001
Never smoked	18.1	20.9	
Former smoker	57.8	59.7	
Current smoker	23.9	19.2	
Unknown	0.2	0.2	
CV risk group (%)			<0.0001
1–No diabetes/no prior CVD	24.6	32.4	
2–Diabetes/no prior CVD	25.4	19.4	
3–Prior CVD	49.9	48.2	
Statin intensity (%)			<0.0001
High	56.5	50.8	
Moderate	39.9	44.9	
Low	3.6	4.3	
eGFR (continuous)	61.7 ± 22.3	63.0 ± 21.7	<0.0001
eGFR (%>60ml/min/1.73m^2^)	51.7	54.8	<0.0001
Systolic blood pressure, mmHg	128.9 ± 16.8	127.6 ± 16.9	<0.0001
Diastolic blood pressure, mmHg	70.0 ± 11.0	68.5 ± 10.9	<0.0001
Total cholesterol, mg/dL	159.3 ± 31.8	141.5 ± 26.6	<0.0001
LDL-C, mg/dL	80.9 ± 26.4	78.1 ± 21.2	<0.0001
HDL-C, mg/dL	36.9 ± 9.9	45.0 ± 14.1	<0.0001
Triglycerides, mg/dL	219.6 ± 68.2	92.8 ± 28.8	<0.0001
CRP^1^	3.2 ± 5.9	3.4 ± 5.9	0.71
HbA1c, %	7.2 ± 1.5	6.7 ± 1.2	<0.0001
Serum CREATININE	1.4 ± 2.3	1.3 ± 1.9	<0.0001
Medical history (%)			
Acute coronary syndrome	19.8	17.6	<0.0001
Coronary revascularization	3.0	2.1	<0.0001
Diabetes	55.0	41.1	<0.0001
Hypertension	93.1	91.6	<0.0001
Ischemic stroke	12.9	13.0	<0.0001
Myocardial infarction	17.5	16.4	<0.0001
Peripheral artery disease	25.6	24.2	<0.0001
Follow-up duration, yrs	4.6 ± 1.6	4.8 ± 1.4	<0.0001

0 Reporting mean +/- SD unless otherwise noted.

1 Missing for 95% of cohort.

### Clinical outcomes

**Figure 2:**
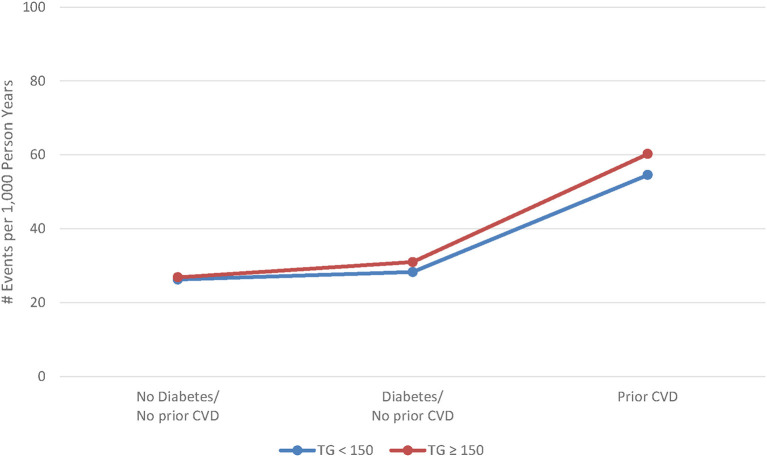
Adjusted rate of major cardiovascular events by cardiovascular risk and triglyceride level.

Veterans with elevated TG levels experienced higher risk of both composite and individual non-fatal CV events during the average 4.8-year follow-up. The overall crude and adjusted (adjusted for age, sex, SBP, eGFR, and weight) composite MACE rate ratios were 1.10 (95% CI 1.09, 1.12) and 1.05 (95% CI 1.03, 1.06), respectively (Table 3). The crude and adjusted composite rate ratios for nonfatal events only were 1.33 (95% CI 1.30, 1.36) and 1.16 (95% CI 1.13, 1.18), respectively. Adjusted rate ratios for non-fatal events ranged from 1.10 (95% CI 1.04, 1.15) to 1.30 (95% CI 1.21, 1.40), while the adjusted rate ratio for cardiovascular death was 0.98 (95% CI 0.96, 1.00). There was little difference between TG groups in both the unadjusted and adjusted risk of MACE outcomes across CV risk groups (Table 4). However, the rate of clinical events increased with elevated CV risk, in that those who had established CVD had a higher event rate as compared to those without diabetes or prior CV event and those with diabetes (Figure 2).

**Table 3:** Crude prevalence, crude and adjusted rate ratios for elevated vs. normal TG comparisons of cardiovascular outcomes.

	**Elevated TG**	**Normal TG**	**Unadjusted rate ratio^a^**	**Adjusted rate ratio^b^**
	**(*n* = 109,195)**	**(*n* = 286,994)**	**(95% CI)**	**(95% CI)**
Composite CV outcome^c^	22,180 (20.3%)	54,762 (19.1%)	1.10 (1.09, 1.12)	1.05 (1.03, 1.06)
Composite non-fatal CV outcome^d^	10,657 (9.8%)	21,906 (7.6%)	1.33 (1.30, 1.36)	1.16 (1.13, 1.18)
Individual CV end points				
Non-fatal MI	6,469 (5.9%)	13,189 (4.6%)	1.34 (1.30, 1.38)	1.16 (1.12, 1.19)
Non-fatal stroke	2,508 (2.3%)	5,623 (2.0%)	1.21 (1.15, 1.27)	1.10 (1.04, 1.15)
Coronary revascularization	1,190 (1.1%)	1,909 (0.7%)	1.69 (1.57, 1.82)	1.30 (1.21, 1.40)
Unstable angina	3,087 (2.8%)	5,541 (1.9%)	1.52 (1.45, 1.59)	1.28 (1.22, 1.34)
Cardiovascular death	13,510 (12.4%)	37,062 (12.9%)	0.98 (0.96, 1.00)	0.98 (0.96, 1.00)

a Rate ratio for each outcome based on generalized linear model with Poisson errors.

b Adjusted rate ratio for each outcome measure controlling for age, sex, SBP, eGFR, and weight. Analysis based on 386,783 subjects with complete data.

c Composite CV outcome was determined as 1^st^ occurrence of any individual CV endpoints.

d Composite non-fatal CV outcome was determined as 1^st^ occurrence of any of non-fatal MI, non-fatal stroke, coronary revascularization, and unstable angina.

**Table 4:** Crude and adjusted rate ratios (95% confidence intervals) for elevated vs. normal TG comparisons of cardiovascular outcomes, stratified by cardiovascular risk group.

	**CV risk group 1** **(No diabetes/no prior CVD)** ***n* = 119,756**	**CV risk group 2** **(Diabetes/no prior CVD)** ***n* = 83,546**	**CV risk group 3** **(Prior CVD)** ***n* = 192,887**
**Composite outcome prevalence**	15,703 (13.1%)	12,044 (14.4%)	49,195 (25.5%)
Elevated TG Group	3,514 (13.1%)	4,150 (14.9%)	14,516 (26.6%)
Normal TG Group	12,189 (13.1%)	7,894 (14.2%)	34,679 (25.1%)
**Unadjusted outcomes^a^**			
Composite CV outcome^b^	1.02 (0.99, 1.06)	1.09 (1.05, 1.14)	1.10 (1.08, 1.13)
Composite nonfatal CV outcome^c^	1.27 (1.18, 1.36)	1.36 (1.27, 1.45)	1.29 (1.26, 1.33)
Individual CV end points			
Nonfatal MI	1.25 (1.14, 1.37)	1.31 (1.21, 1.42)	1.31 (1.26, 1.35)
Nonfatal stroke	1.15 (1.01, 1.31)	1.38 (1.24, 1.55)	1.13 (1.07, 1.20)
Coronary revascularization	–	–	1.69 (1.57, 1.82)
Unstable angina	1.36 (1.17, 1.58)	1.58 (1.37, 1.82)	1.48 (1.41, 1.55)
Cardiovascular Death	0.96 (0.92, 1.00)	1.00 (0.95, 1.04)	0.97 (0.95, 1.00)
**Adjusted Outcomes** ^d^			
Composite CV outcome^2^	0.98 (0.94, 1.01)	1.05 (1.01, 1.09)	1.05 (1.03, 1.07)
Composite nonfatal CV outcome	1.07 (1.00, 1.15)	1.20 (1.12, 1.27)	1.15 (1.11, 1.18)
Individual CV end points			
Nonfatal MI	1.06 (0.96, 1.16)	1.15 (1.06, 1.26)	1.16 (1.12, 1.20)
Nonfatal stroke	1.02 (0.89, 1.16)	1.27 (1.13, 1.42)	1.05 (0.99, 1.12)
Coronary revascularization^e^	–	–	1.30 (1.21, 1.40)
Unstable angina	1.10 (0.94, 1.28)	1.36 (1.18, 1.57)	1.28 (1.21, 1.34)
Cardiovascular Death	0.98 (0.93, 1.02)	1.01 (0.96, 1.05)	0.97 (0.95, 1.00)

a Rate ratio for each outcome based on generalized linear model with Poisson errors.

b Composite CV outcome was determined as 1^st^ occurrence of any individual CV endpoints.

c Composite non-fatal CV outcome was determined as 1^st^ occurrence of any of non-fatal MI, non-fatal stroke, coronary revascularization, and unstable angina.

d Adjusted for age, sex, SBP, eGFR, and weight. Analysis based on 386,783 subjects with complete data.

e All coronary revascularizations occurred in CV Risk Group 3.

Additionally, the relationship between TG level and the MACE outcome did not differ by race or statin intensity. Treating TG level as a time-varying exposure did not change the relationship between TG level and the outcomes of interest.

## Discussion

The principal findings of this large, retrospective assessment of U.S. veterans with either established CHD or with risk factors for CHD were that patients with elevated baseline TG levels (150–499 mg/dL) demonstrated higher incident non-fatal CV event rates during an average 4.8-year follow-up, despite well-controlled LDL-C levels (40–100 mg/dL), as compared with patients with normal TG levels at baseline. The overall crude non-fatal CV event rate ratio for the composite outcome was 1.33 (95% CI 1.30, 1.36) and, after adjustment for age, sex, baseline systolic blood pressure, eGFR, and weight, the composite non-fatal CV event ratio was 1.16 (95% CI 1.13, 1.18). When adjusting outcomes for the 3 prespecified CV risk groups, there was—as expected— a higher event rate among subjects with established ASCVD as compared with patients with no CV risk factors or those with diabetes. We were able to show an increased risk associated with elevated TG on both crude and adjusted non-fatal CV events but not on fatal CV events, though this increased risk did not meaningfully differ by CV risk group. Though this association of increased MACE was observed for most non-fatal events, we could not demonstrate any between-group difference in CV mortality.

We must recognize that the concept of residual CV risk should not be so narrowly viewed as being solely attributed to LDL-C mediated mechanisms alone. In this regard, it is important to emphasize that hypertriglyceridemia is a highly prevalent lipid disorder in the adult population. According to the AHA scientific statement on triglycerides and CVD (45), 31% of adults age ≥ 20 years had triglycerides ≥ 150 mg/dl in the US-NHANES survey (1999–2008). These numbers are currently likely to be even higher given the continued escalation globally of the triple epidemics of obesity, metabolic syndrome, and type 2 diabetes. In addition to the estimated ~30 million adults with diabetes in the U.S. and more than 415 million worldwide (46, 47), there is a 3-fold higher rate of pre-diabetes/insulin resistance and cardio-metabolic syndrome as compared with established diabetes. Thus, the cardinal manifestations of metabolic syndrome are protean and include the dyslipidemic phenotype of elevated levels of TG, low HDL-C, and small dense LDL-C particles along with clinical features of visceral abdominal obesity and non-alcoholic fatty liver disease. These findings underscore how insulin resistance plays such a major role in mediating the pathogenesis of metabolic syndrome (48–50). While there are other notable causes of residual cardiovascular risk, including thrombotic, glycemic, metabolic, and inflammatory pathways, these non-lipid causes of residual CV risk are beyond the scope of this paper.

As noted previously, a substantial residual risk of CV events, even after initiation of intensive statin therapy (13, 51) to lower LDL-C in clinical trials, has prompted re-assessment for the role of other lipoproteins contributing to subsequent clinical events in high-risk individuals. Meta-analyses of long-term prospective studies have reported an association between elevated TG and CHD (14, 52–54), although attenuated after adjustments for HDL-C, as these two lipid fractions are highly inversely correlated.

Recently, two very large multi-year observational studies have confirmed the triglyceride-associated increase in CV events in statin treated patients even after multivariate adjustments including LDL-C, HDL-C, and other risk factors. In the study by Nichols et al. (42) who followed 2,702 subjects with TG 200–499 mg/dl, and 14,481 subjects with TG <150 mg/dl, there was a 20% increase in MI (*p* = 0.045), an 18% increase in coronary revascularization (*p* = 0.045), and a 7% increase in composite outcome (*p* = 0.127) including total mortality. Similarly, Toth et al. (43) performed a retrospective insurance claims, propensity–matched analysis of subjects derived from the Optum Health database with high or normal TG levels as defined above by Nichols and colleagues (10,990 in each group). There was a 35% increase in major CV events, including CV death, (*p* < 0.001), a 15% increase in total healthcare costs (*p* < 0.001), and a 17% increase in inpatient hospital stays (*p* < 0.001). The present study is the largest observational database of clinical outcomes to date among patients with elevated baseline TG and well-controlled LDL-C levels on statins, the results of which are concordant with similar observational findings cited above (42, 43). In addition, the results of this large VA analysis of clinical outcomes among veterans with elevated vs. normal TG levels at baseline clearly highlight the prevalence of this dyslipidemia phenotype.

It is abundantly clear that the prevalence of elevated TG will continue to escalate in all regions of the world as the insidious epidemics of type 2 diabetes, metabolic syndrome, and obesity continue to accelerate globally. While the observational epidemiology supporting the association between elevated baseline TG levels and low HDL-C levels with increased incident rates of cardiovascular events is strong and robust for both men and women, (4) they do not provide unconfounded estimates of causality. By contrast, there is compelling scientific evidence from recent genetic studies using Mendelian randomization which provide a causal role for hypertriglyceridemia, rather than low baseline levels of HDL-C, in contributing directly to elevated ASCVD risk. In one such large multivariable Mendelian randomization study involving approximately 20,000 myocardial infarction (MI) cases and 50,000 controls, for every 1 SD increase in baseline TG levels, there was a significant 54% increase in risk for coronary heart disease (21). It is also notable that TG levels in plasma may be influenced by multiple genes as well as environmental factors (55). Additionally, several genetic studies have likewise shown that genetically lower TG concentrations irrespective of the mechanism result in a lower risk of incident ASCVD events, (14, 56–58) while conversely, there was no association observed with genetically lower levels of HDL-C and incident ASCVD event rates (59). In another very large Mendelian randomization study involving 654,783 participants, triglycerides were shown to be an independent causal factor for ASCVD, with an effect that appeared to be modulated by ApoB 100 levels (20). This latter finding likely reflects the fact that triglycerides are carried in very low-density lipoproteins (VLDL) and VLDL remnants, such as small VLDL particles and intermediate-density lipoproteins, all of which contain apoB100. Triglyceride-enriched lipoproteins correlate highly with increased risk for ASCVD events (60, 61). In addition to their triglyceride cargo, remnant lipoproteins may be proatherogenic because they also carry cholesterol and are proinflammatory (62, 63). While prior placebo-controlled trials in the statin era that tested the co-administration of fibrates or niacin have failed to demonstrate incremental ASCVD event reduction after “optimally controlled” LDL-C levels have been achieved with statins, (29) it is notable that therapies such as fibrates (in particular gemfibrozil) and niacin also lower LDL-C and ApoB with benefits proportional to the reductions in ApoB (64).

### Limitations

As with all observational retrospective analyses, there are well-recognized confounders that limit the generalizability of these findings to broader populations of unselected patients. Veterans comprise a disproportionately large male demographic, and these findings may not apply to populations that include a larger percentage of women. This cohort also includes a large majority white subjects. In addition, event ascertainment was based on coding (ICD-9) derived from the large VA CDW and the EHR; there was no independent chart review to assess the actual occurrence of clinical events. We do not have data to corroborate patient adherence to dyslipidemic therapy during the follow-up period, nor do we have knowledge of the use of hospitalizations occurring or medications prescribed outside of the VA healthcare system, or the use of over-the-counter medications that could potentially alter the lipid profile over time. Finally, we did not assess serial measurements of lipid profiles over time, nor do we know that on-treatment LDL-C levels remained in the range of 40–100 mg/dL during long-term follow-up.

## Conclusion

In this large cohort of U.S. veterans, those with elevated TG levels showed an increase in non-fatal CV events despite well-controlled LDL-C on statins compared with veterans whose baseline TG was in a normal range. These data do add to the growing body of scientific information that elevated TG levels are prevalent in the U.S. veteran population and helps identify an important subset of individuals at high residual risk for recurrent CV events for whom therapies such as icosapent ethyl may be ideally suited to improve event-free survival.

## Data availability statement

The data analyzed in this study is subject to the following licenses/restrictions: Data are owned by the VA and may only be shared through approved data use agreements. Requests to access these datasets should be directed to sarah.leatherman@va.gov.

## Ethics statement

The studies involving human participants were reviewed and approved by VA Boston Healthcare System Institutional Review Board and Research and Development Committee. Written informed consent for participation was not required for this study in accordance with the national legislation and the institutional requirements.

## Author contributions

SL, RF, KH, CG, SP, DB, PT, and WB: study conception and design. CH: data collection and analysis. SL, RF, CH, and KH: interpretation of results. SL, RF, KH, DB, PT, and WB: draft manuscript preparation. All authors have approved the final manuscript.

## Conflict of interest

Author CG was employed by Lexicon Pharmaceuticals. Author SP was employed by Amarin Pharma Inc. Author PT has served as a speaker and consultant for Amarin Pharma Inc. Authors DB and WB have received research funds from Amarin Pharma Inc. The authors declare that this study received funding from Amarin Pharma, Inc. The funder had the following involvement in the study: input into study protocol design, review of results, review of manuscript.

## Publisher's note

All claims expressed in this article are solely those of the authors and do not necessarily represent those of their affiliated organizations, or those of the publisher, the editors and the reviewers. Any product that may be evaluated in this article, or claim that may be made by its manufacturer, is not guaranteed or endorsed by the publisher.
